# A duplex real-time PCR assay for the detection and quantification of avian reovirus and *Mycoplasma synoviae*

**DOI:** 10.1186/s12985-015-0255-y

**Published:** 2015-02-12

**Authors:** Li Huang, Zhixun Xie, Liji Xie, Xianwen Deng, Zhiqin Xie, Sisi Luo, Jiaoling Huang, Tingting Zeng, Jiaxun Feng

**Affiliations:** Guangxi Key Laboratory of Animal Vaccines and New Technology, Guangxi Veterinary Research Institute, Nanning, 530001 PR China; College of Life Science and Technology, Guangxi University, Nanning, 530004 PR China

**Keywords:** Duplex real-time PCR assay, Avian reovirus, *Mycoplasma synoviae*

## Abstract

**Background:**

Infectious arthritis in broilers represents an economic and health problem, resulting in severe losses due to retarded growth and downgrading at the slaughterhouse. The most common agents associated with cases of infectious arthritis in poultry are avian reovirus (ARV) and *Mycoplasma synoviae* (MS). The accurate differentiation and rapid diagnosis of ARV and MS are essential prerequisites for the effective control and prevention of these avian pathogens in poultry flocks. This study thus aimed to develop and validate a duplex real-time PCR assay for the simultaneous detection and quantification of ARV and MS.

**Methods:**

Specific primers and probes for each pathogen were designed to target the special sequence of the ARV σC gene or the MS phase-variable surface lipoprotein hemagglutinin (*v*lhA) gene. A duplex real-time PCR assay was developed, and the reaction conditions were optimized for the rapid detection and quantification of ARV and MS.

**Results:**

The duplex real-time PCR assay was capable of ARV- and MS-specific detection without cross-reaction with other non-targeted avian pathogens. The sensitivity of this assay was 2 × 10^1^ copies for a recombinant plasmid containing ARV σC or MS *v*lhA gene, and 100 times higher than that of conventional PCR. This newly developed PCR assay was also reproducible and stable. All tested field samples of ARV and/or MS were detectable with this duplex real-time PCR assay compared with pathogen isolation and identification as well as serological tests.

**Conclusion:**

This duplex real-time PCR assay is highly specific, sensitive and reproducible and thus could provide a rapid, specific and sensitive diagnostic tool for the simultaneous detection of ARV and MS in poultry flocks. The assay will be useful not only for clinical diagnostics and disease surveillance but also for the efficient control and prevention of ARV and MS infections.

## Background

Infectious arthritis in broilers represents an economic and health problem, resulting in severe losses due to retarded growth and downgrading at the slaughterhouse. The most common agents associated with cases of infectious arthritis in poultry are avian reovirus (ARV) and *Mycoplasma synoviae* (MS). ARV belongs to the Orthoreovirus genus, one of nine genera of the Reoviridae family [1,2]. ARV infection is associated with several disease syndromes and especially viral arthritis/tenosynovitis in chickens [3,4]. Meanwhile, MS is a common pathogen found in turkeys and chickens that causes diseases of the respiratory tract, urogenital tract and joints and impairs growth [5,6]. Mixed infections of ARV and MS have occurred in poultry flocks worldwide and have similar clinical signs, including severe immunosuppression, arthritis, depression, retarded growth, weight loss and decreased egg production. Bradbury [7] and Reck [8] also found that in chickens, a synergistic relationship exists between ARV and MS, which causes much more severe clinical signs and pathological lesions than the additive effects of these two pathogens alone do. The main feature of possible economic importance in ARV and MS infection is the incidence of decreased egg production and fertility, sternal bursitis leading to carcass downgrading and leg abnormalities related to condemnation of broilers. As the elimination of lesioned carcasses at the slaughterhouse is important [3,9], the rapid and efficient detection and diagnosis of ARV and MS are essential prerequisites for the effective control and prevention of these avian pathogens in poultry flocks.

The current methods for ARV and MS detection include serological assays; pathogen isolation and identification; and molecular detection methods, such as single PCR and multiplex PCR [10-13]. However, these assays are laborious and time consuming, have limited specificity and sensitivity, and require post-amplification procedures. Real-time PCR assays for the specific identification of a target sequence by fluorescent probes can overcome these limitations and provide distinct advantages, such as a shorter detection time, improved sensitivity and specificity, simplified closed-tube procedures and the potential for pathogen screening and surveillance in commercial poultry flocks [14-16].

Therefore, the present study developed and validated a duplex real-time PCR assay for the differential diagnosis and quantitative detection of ARV and MS.

## Materials and methods

### Ethics statement

This study was performed in strict accordance with the recommendations in the Guide for the Care and Use of Laboratory Animals of the Guangxi Veterinary Research Institute. Additionally, the Animal Care and Use Committee of the Guangxi Veterinary Research Institute approved all procedures involving the use of animals, and all efforts were made to minimize animal suffering.

### Pathogens and construction of recombinant plasmids

DNA was first extracted from MS samples as described previously [17], and total RNA was extracted from ARV samples using TRIzol reagent (Life Technologies, Carlsbad, CA, USA) following the manufacturer’s instructions. Next, cDNA was generated as described previously [18] and used as a template for a duplex real-time PCR assay. Target gene fragments from the ARV σC gene or the MS phase-variable surface lipoprotein hemagglutinin (*v*lhA) gene were then amplified with the primers listed in Table 1 and inserted into the pMD18-T vector (TaKaRa, Dalian, China). Subsequently, the constructed plasmids were transformed into DH5α *Escherichia coli*. The recombinant plasmids carrying each target gene were confirmed by sequencing and were used as positive standards for ARV and MS. The copy number of each positive-standard plasmid was calculated as described previously [19].

**Table 1:** **Specific primers used to clone ARV and MS specific genes**

**Primer name**	**Primer sequence**	**Amplicon length**	**Target gene**
ARV C703F	5’-TGTGGATCCATGGCGGGTCTCAAT-3’	981 bp	σC gene
ARV C703R	5’-CCGGAATTCTAAGGTGTCGATGCC-3’		
MS *v*lhA F2	5’-CTGTTATAGCAATTTCATGTGGTG-3’	283 bp	phase-variable surface lipoprotein hemagglutinin (*v*lhA)
MS *v*lhA R2	5’-TGTTGTAGTTGCTTCAACTTGTCT-3’		

### Oligonucleotide primers and DNA probes for duplex real-time PCR

DNASTAR software (DNASTAR Inc., Madison, WI, USA) was used to confirm the highly conserved regions in the ARV and MS genomes, and Primer Express 3.0 software (Applied Biosystems, Foster City, CA, USA) was used to design the primers and probes listed in Table 2 for ARV and MS, based on their highly conserved regions. The cross-reactivity of the oligonucleotides was assessed by BLAST analysis. Both sets of primers and probes were synthesized by TaKaRa (Dalian, China).

**Table 2:** **Primers and probes used for the duplex real-time PCR assay**

**Primer/probe name**	**Primer/probe sequence**	**Target gene**	**Amplicon length**
ARV F	5’-CGTTCCCTGTGGACGTATCA-3’	σC	69 bp
ARV R	5’-GAGTACACCCCATACGCTTGGT-3’		
ARV P	5’-(FAM) TCACCCGCGATTCTGCGACTCAT (Eclipse)-3’		
MS-F	5’-ATAGCAATTTCATGTGGTGATCAA-3’	*v*lhA	143 bp
MS-R	5’**-**TGGATTTGGGTTTTGAGGATTA-3’		
MS-P	5’-(ROX) CAGCACCTGAACCAACACCTGGAA (Eclipse)-3’		

### Duplex real-time PCR assay for simultaneous MS and ARV detection

The duplex real-time PCR was performed in a 20-μl volume. The reaction mixture included 1× real-time PCR Premix (Perfect Real Time PCR Kit, TaKaRa, Dalian, China); 0.3 μM ARV F, ARV R and ARV P primers; 0.3 μM MS F, MS R and MS P primers; and 2 μl of positive-plasmid template. Sterilized H_2_O was added to bring the final volume to 20 μl. The protocol for the reaction was 95°C for 30 sec; 45 cycles of 95°C for 10 sec and 60°C for 30 sec; and, finally, 40°C for 5 sec. The fluorescence was measured at the end of each 60°C incubation. The data analysis was performed using Light Cycler 2.0 system software (Roche, Molecular Biochemical, Mannheim, Germany).

### Conventional RT-PCR and PCR

Conventional RT-PCR for ARV amplification and conventional PCR for MS amplification were performed. The PCR mixture contained 2× Premix Taq (TaKaRa, Dalian, China), 0.4 μM forward primer or reverse primer, 2 μl of template and sterilized H_2_O to bring the final reaction volume to 25 μl. The conditions for PCR were 95°C for 5 min; 72°C for 7 min; and three-step cycling 35 times at 95°C for 30 sec, 60°C for 30 sec and 72°C for 30 sec. The PCR product was run on a 2% agarose gel at 80 V for 45 min and visualized on a molecular imager Gel Doc XR+ imaging system with Image Lab software (Bio-Rad, Life Science Research, Hercules, CA, USA).

### Specificity and sensitivity of the duplex real-time PCR assay

To assess the specificity of the assay, DNA from *Mycoplasma gallisepticum* (MG), *Mycoplasma iowae* (MI) and *Mycoplasma meleagridis* (MM) were extracted as described previously [17]. Additionally, cDNA was generated from total RNA that was extracted from cases of newcastle disease virus (NDV), infectious bursal disease virus (IBDV), avian infectious bronchitis virus (AIBV), the H9 subtype of the avian influenza virus (AIV), Marek’s disease virus (MDV), reticuloendotheliosis virus (REV), and avian leukosis virus (ALV) using TRIzol reagent (Life Technologies, Carlsbad, CA, USA) following the manufacturer’s instructions. The DNA and cDNA were mixed together in equal concentrations as the templates and were subjected to the optimized duplex real-time PCR assay to detect ARV and MS. The sensitivity of the duplex real-time PCR assay was determined using serial 10-fold dilutions (10^1^-10^8^ copies/μl) of positive-plasmid combinations carrying the MS and ARV target genes as templates. These results were compared with the results of conventional PCR. To generate a standard curve for ARV and MS, the threshold cycle (Ct) of these standard dilutions was plotted against the log value of the copy number of the corresponding standard plasmid.

### Reproducibility and interference tests of the duplex real-time PCR assay

To assess the intra- and inter-assay reproducibility, three samples with the same concentration (10^8^ copies/μl) of the MS or ARV target gene were assessed using the duplex real-time PCR assay. The same experiments were repeated in triplicate every two days for seven days. The reproducibility was then analyzed based on the standard deviation (SD) and the coefficient of variability (CV) of the Ct average. To determine the reaction efficiency interference, different concentrations of positive plasmids carrying the ARV or MS target gene (10^8^ and 10^1^ copies/μl, respectively) were analyzed using the duplex real-time PCR assay.

### Duplex real-time PCR analysis of field samples

All field samples, such as joints and joint contents, were collected from chicks and broilers exhibiting clinical signs of MS or ARV infections and were used to validate the duplex real-time PCR assay. The results were compared with those of traditional diagnostic methods, such as pathogen isolation and identification and serological tests.

## Results

### Specificity test

**Figure 1:**
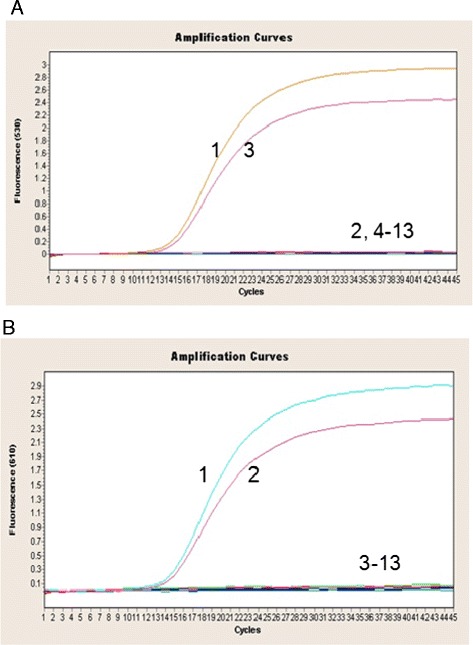
**Specificity of the duplex real-time PCR assay for ARV and MS. (A)** Specificity of the duplex real-time PCR assay for ARV. 1. MS + ARV, 2. MS, 3. ARV, 4. IBDV, 5. NDV, 6. AIBV, 7. MDV, 8. H9 subtype of AIV, 9. REV, 10. ALV, 11. MG, 12. MI, 13. MM, 14. negative control. **(B)** Specificity of the duplex real-time PCR assay for MS. 1. MS + ARV, 2. MS, 3. ARV, 4. IBDV, 5. NDV, 6. AIBV, 7. MDV, 8. H9 subtype of AIV, 9. REV, 10. ALV, 11. MG, 12. MI, 13. MM, 14. negative control.

The specificity of the duplex real-time PCR assay was verified by examining DNA/cDNA from different samples infected with different pathogens. As shown in Figure 1 and Table 3, the duplex real-time PCR assay was able to detect and differentiate ARV and MS independently and simultaneously. In contrast, the other avian pathogens (NDV, IBDV, AIBV, AIV, MDV, REV, ALV, MG, MI and MM) were not detected using the duplex real-time PCR assay. When samples were co-infected with both ARV and MS, unique amplification curves were simultaneously produced in the 530 nm and 610 nm channels, whereas a single amplification curve was observed in the 530 nm or 610 nm channel when samples were infected with only ARV or MS, respectively. Thus, the specificity of the duplex real-time PCR assay was 100%, with no detectable fluorescent signals for other avian pathogens or negative controls.

**Table 3:** **Pathogens used and Ct values of the duplex real-time PCR assay’s specificity**

**Pathogen**	**Number of samples**	**Ct values of duplex real-time PCR assay**	
		**MS**	**ARV**
NDV	1	Negative	Negative
H9 subtype of AIV	1	Negative	Negative
AIBV	1	Negative	Negative
IBDV	1	Negative	Negative
MG	1	Negative	Negative
MM	1	Negative	Negative
MI	1	Negative	Negative
MDV	1	Negative	Negative
pMD18-T-ARV	1	Negative	15.58
pMD18-T-MS	1	15.31	Negative
REV	1	Negative	Negative
ALV	1	Negative	Negative

### Sensitivity test

**Figure 2:**
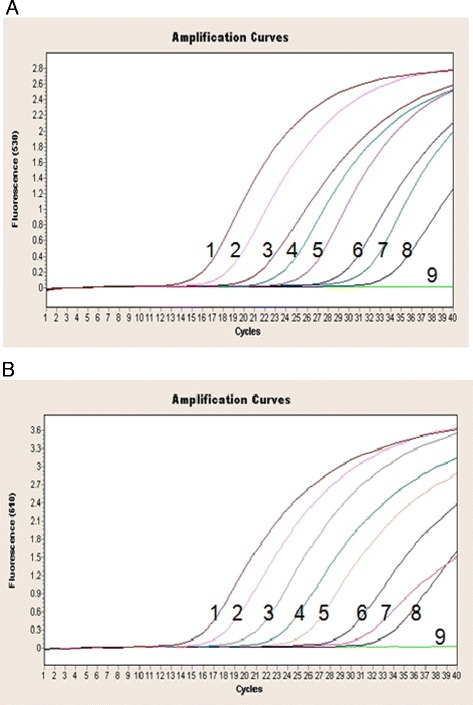
**Sensitivity of the duplex real-time PCR assay for ARV and MS. (A)** Sensitivity of the duplex real-time PCR assay for ARV. 1. 2 × 10^8^ copies, 2. 2 × 10^7^ copies, 3. 2 × 10^6^ copies, 4. 2 × 10^5^ copies, 5. 2 × 10^4^ copies, 6. 2 × 10^3^ copies, 7. 2 × 10^2^ copies, 8. 2 × 10^1^ copies, 9. negative control. **(B)** Sensitivity of the duplex real-time PCR assay for MS. 1. 2 × 10^8^ copies, 2. 2 × 10^7^ copies, 3. 2 × 10^6^ copies, 4. 2 × 10^5^ copies, 5. 2 × 10^4^ copies, 6. 2 × 10^3^ copies, 7. 2 × 10^2^ copies, 8. 2 × 10^1^ copies, 9. negative control.

**Figure 3:**
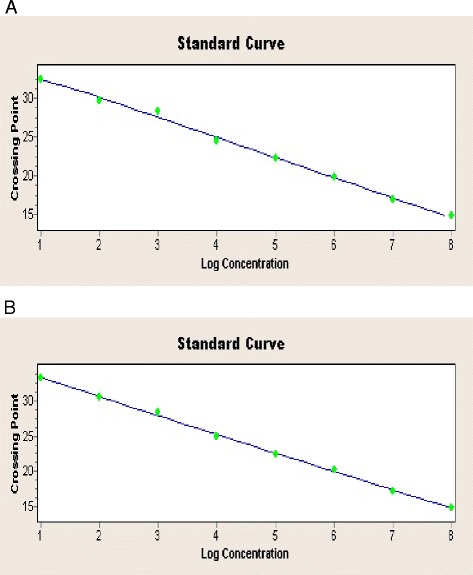
**ARV and MS standard curves. (A)** ARV standard curve. **(B)** MS standard curve.

**Figure 4:**
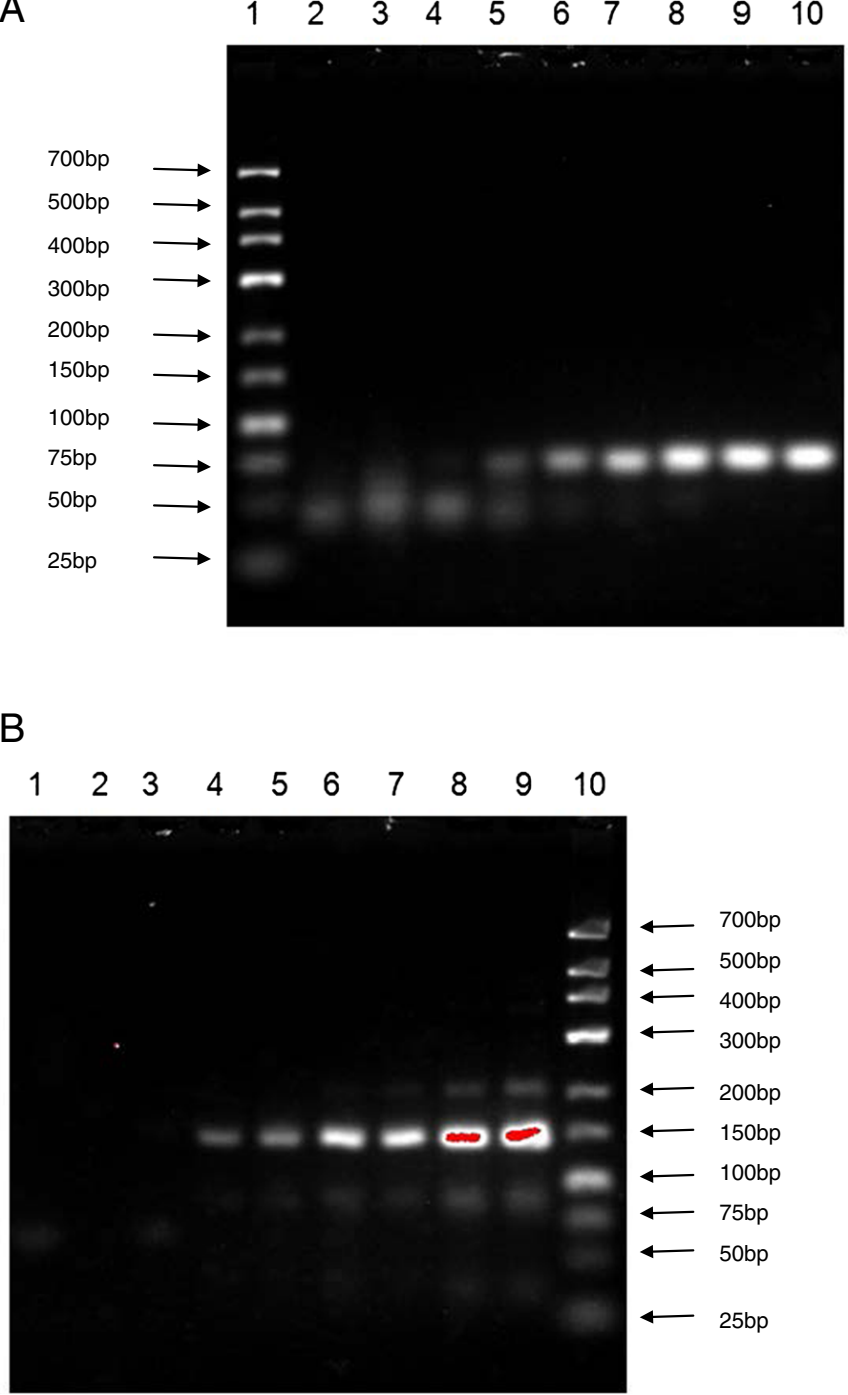
**Sensitivity of conventional PCR for ARV and MS. (A)** Sensitivity of conventional PCR for ARV. 1. low ladder, 2. negative control, 3. 2 × 10^1^ copies, 4. 2 × 10^2^ copies, 5. 2 × 10^3^ copies, 6. 2 × 10^4^ copies, 7. 2 × 10^5^ copies, 8. 2 × 10^6^ copies, 9. 2 × 10^7^ copies, 10. 2 × 10^8^ copies. **(B)** Sensitivity of conventional PCR for MS. 1. negative control, 2. 2 × 10^1^ copies, 3. 2 × 10^2^ copies, 4. 2 × 10^3^ copies, 5. 2 × 10^4^ copies, 6. 2 × 10^5^ copies, 7. 2 × 10^6^ copies, 8. 2 × 10^7^ copies, 9. 2 × 10^8^ copies, 10. low ladder.

The sensitivity of the duplex real-time PCR assay was verified by testing 10^1^-10^8^ copies/μl of recombinant plasmids carrying the ARV or MS target gene. In Figure 2, the ARV amplification curves are shown in the 530 nm channel (Figure 2A), and the MS amplification curves are shown in the 610 nm channel (Figure 2B). Moreover, the standard curves for ARV and MS are shown in Figure 3, and the Ct values are listed in Table 4. The results revealed that even with a template amount as low as 2 × 10^1^ copies, the ARV or MS target gene was still detectable. In contrast, the detection limit of the conventional PCR template was 2 × 10^3^ copies for ARV and MS (Figure 4), which is 100 times lower than that of the duplex real-time PCR assay. Thus, the duplex real-time PCR assay is highly sensitive.

**Table 4:** **Ct values from the serial dilution of positive plasmids**

**Tenfold dilution**	**2 × 10** ^**8**^ **copies**	**2 × 10** ^**7**^ **copies**	**2 × 10** ^**6**^ **copies**	**2 × 10** ^**5**^ **copies**	**2 × 10** ^**4**^ **copies**	**2 × 10** ^**3**^ **copies**	**2 × 10** ^**2**^ **copies**	**2 × 10** ^**1**^ **copies**
ARV (Ct)	14.86	17.21	20.2	22.49	24.9	28.36	30.45	33.18
MS (Ct)	14.79	16.83	19.77	22.18	24.49	28.24	29.72	32.41

### Reproducibility and interference tests

**Figure 5:**
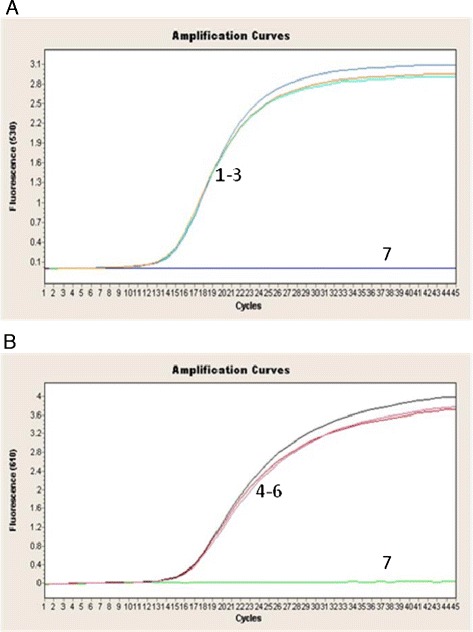
**Reproducibility of the duplex real-time PCR assay for ARV (A) and MS (B).** 1–3. ARV, 4–6. MS, 7. negative control.

The reaction of reproducibility was determined by testing three samples of the same concentration at the same time points and was assessed using the SD and CV of the Ct values for each sample. The intra-assay reproducibility results are shown in Figure 5, and the inter-assay reproducibility results are listed in Table 5. The CV values were 1.61% for ARV and 1.89% for MS. These data indicate that the findings produced by the duplex real-time PCR assay are reproducible.

**Table 5:** **Reproducibility of the duplex real-time PCR assay for ARV and MS**

**Pathogen**	**Ct values of same samples at different time points**				
	**Day 1**	**Day 4**	**Day 7**	**SD**	**CV**
ARV	15.23/1 × 10^8^	15.4/1 × 10^8^	15.36/1 × 10^8^	0.24	1.61%
MS	14.96/1 × 10^8^	14.7/1 × 10^8^	15.09/1 × 10^8^	0.28	1.89%

Because the presence of other templates may affect the amplification efficiency of a PCR assay, we tested the influence of mixtures of different concentrations on reaction sensitivity. In particular, a combination of a high concentration (10^8^ copies/μl) and a low concentration (10^2^ copies/μl) of positive plasmids carrying the ARV or MS target gene was used in an interference test (Table 6). The results indicated that there were no systematic deviations in the amplification curves of the mixed templates compared with those of the single template; moreover, the CV value was less than 3% (data not shown). The results suggest that the newly developed duplex real-time PCR assay is stable.

**Table 6:** **Samples used in the interference test**

**Pathogen**	**Sample 1**	**Sample 2**	**Sample 3**	**Sample 4**	**Sample 5**	**Sample 6**
ARV	2 × 10^8^ copies	2 × 10^2^ copies	2 × 10^8^ copies	--	2 × 10^2^ copies	--
MS	2 × 10^2^ copies	2 × 10^8^ copies	--	2 × 10^8^ copies	--	2 × 10^2^ copies

### Field samples

The detection results of the duplex real-time PCR assay for 40 field samples are listed in Table 7 and the results were confirmed by pathogen isolation and identification or serological tests. The ARV detection rate was 7.5%, and the MS detection rate was 5%. Additionally, the range of Ct values for ARV was 15.29-34.42, and the range of Ct values for MS was 13.53-30.68. Thus, the results of this new assay were comparable with the results of other detection approaches.

**Table 7:** **Detection of field samples using the duplex real-time PCR assay**

	**ARV**	**MS**
Positive samples/total samples	3/40	2/40
Positive rate	7.5%	5%

## Discussion

Both MS and ARV can cause similar clinical signs and lesions and may be present as co-infections in chickens and other avian species, which can lead to huge economic losses [20]. In this paper, we developed a duplex real-time PCR assay and described its use for the rapid, sensitive and accurate quantitative detection of ARV and MS.

The primary advantage of this duplex real-time PCR assay is the simultaneous detection and differentiation of ARV and MS. By using unique primer and probe sets within the highly conserved gene regions of ARV and MS, this duplex real-time PCR assay is readily able to detect and differentiate these pathogens via one reaction. Furthermore, this assay is optional and can be utilized as a single-target assay or combined into duplex assays, without impacting the quality of the results. Specifically, duplexing reduces the expense of reagents and the required time for analysis, and the single-target assay makes this assay adaptable to circumstances that may not require the simultaneous detection of these two pathogens for diagnostic purposes. These advantages greatly facilitate clinical application, which is an important criterion for the usefulness of a diagnostic assay for the early surveillance and prevention of diseases [21].

For a method of pathogen detection to be used as a clinical diagnostic tool, sensitivity is a key criterion [22,23]. Using the newly developed assay, as few as 2 x 10^1^ copies could be detected for both ARV and MS, which was more sensitive than the results of a duplex real-time PCR assay reported by Sprygin [24] and the results of the multiplex PCR performed by Reck [11]. Moreover, during detection with mixed samples (other non-targeted pathogens) and field samples, the specificity of this new assay was comparable with that of traditional methods, such as pathogen isolation and identification and serological tests. Therefore, this duplex real-time PCR assay with higher sensitivity rates could be promising as a tool for rapid clinical differentiation and diagnosis at the early stage of ARV and/or MS infection.

Another distinct feature of this duplex real-time PCR assay is the short turn-around time for the results. In the present study, the results for ARV and MS infections were obtained within 2 h with this duplex real-time PCR assay, which is very important for rapid diagnosis, especially during emergent disease outbreaks. Furthermore, the obtained results could be directly visualized on a computer connected to the real-time PCR station. Compared with the conventional diagnostic approaches for ARV and MS infections (and even single and multiplex PCRs [25,26]), this assay does not require additional unique equipment or specialized labor. This method also minimizes post-amplification procedures, such as electrophoresis and UV visualization, which are time consuming. As compared to recently developed isothermal methods for ARV or MS detection, including loop-mediated isothermal amplification [27,28] and cross-priming amplification [29], for which there is no need for expensive equipment except a water bath, the main drawback of the duplex real-time PCR assay is the absolute need for the thermal cycle. However, the method capability of simultaneous detection for ARV and MS highlights its importance and great value for the rapid detection of ARV and MS infections in the laboratory.

Considering the high cost of probe synthesis and the possibility of different genotypes as well as variant or vaccine strains of ARV or MS, the development of new technology or novel reagents for probe synthesis and the design of more primers based on more highly conserved regions of the ARV and MS genomes would be necessary to investigate further modification and optimization of this new assay.

## Conclusion

In this study, we developed a rapid, specific and sensitive duplex real-time PCR assay for the simultaneous detection of ARV and MS. Based on its speed and sensitivity, this newly developed assay could be useful not only for the clinical diagnosis of ARV and MS infections but also for the control and prevention of these infections.
